# Supplementary material for: Illuminating cellular and extracellular vesicle-mediated communication via a split-Nanoluc reporter in vitro and in vivo
Source: Cell Rep Methods. 2023 Feb 21;3(2):100412. doi: 10.1016/j.crmeth.2023.100412 (PMC10014296; doi:10.1016/j.crmeth.2023.100412)
Supplement: Document S1. Figures S1 and S2 [file mmc1.pdf]

**Cell Reports Methods, Volume *3***

# Supplemental information

# Illuminating cellular and extracellular vesicle-mediated communication via a split-Nanoluc reporter *in vitro* and *in vivo*

**Thomas S. van Solinge, Shadi Mahjoum, Stefano Ughetto, Alessandro Sammarco, Marike L.D. Broekman, Xandra O. Breakefield, and Killian P. O'Brien**

Supplemental Figure 1: Quantification of cellular communication with the split-Nanoluc construct. Related to Figure 1.

A.

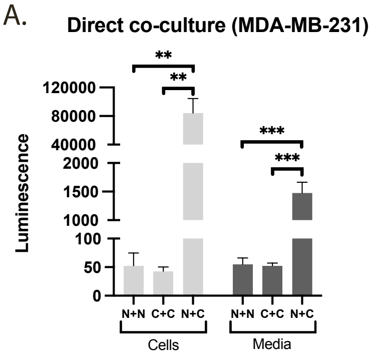


B.

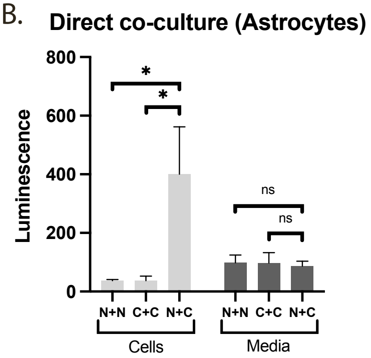


A) Direct co-culture of MDA breast cancer cells transduced with N65 or 66C constructs. Student's independent t-test, **: p<0.01, ***: p<0.001. Related to Figure 1

B) Direct co-culture of astrocytes transduced with either N65 of 66C construct. Student's independent t-test, ns: not significant, *: p<0.05. Related to Figure 1

C.

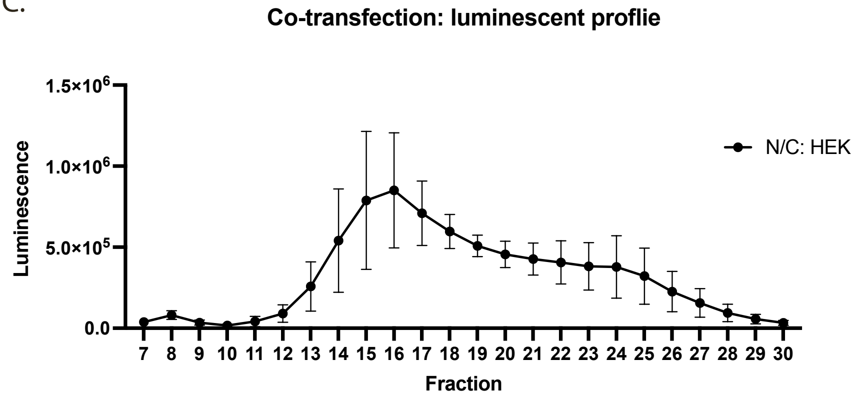


C) Media from HEK cells transfected with both N65 and 66C constructs separated via size exclusion chromatography. FMZ was added and signal recorded after 3 minutes. Related to Figure 1

Supplemental Figure 2: Extracellular vesicle mediated communication with the split-Nanoluc construct. Related to Figure 2.

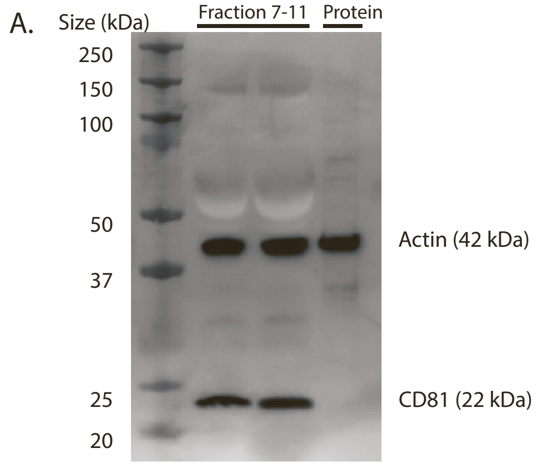


A) Western Blot of EV fractions and protein isolated from HEK293T cells. Stained for Actin and CD81. kDA: kilo Dalton. Related to Figure 2

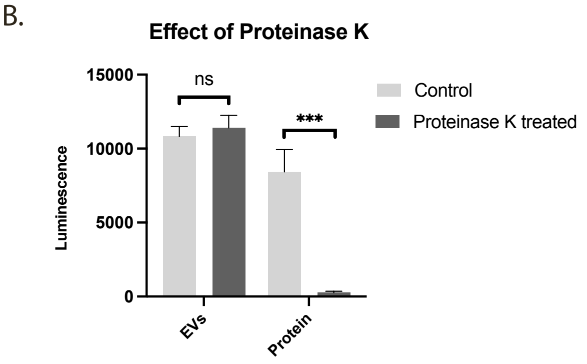


B) The effect of proteinase K treatment on the luminescent signal in EV and protein fractions. Student's independent t-test, ns: not significant, ***: p<0.001. Related to Figure 2.

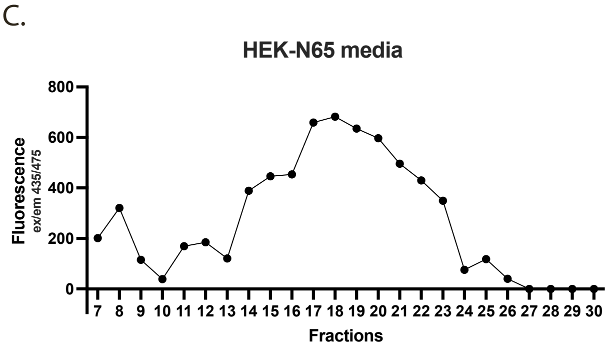


C) Fluorescent signal in different fractions derived via size exclusion chromatography from HEK cells transfected with the N65 construct. Related to Figure 2.

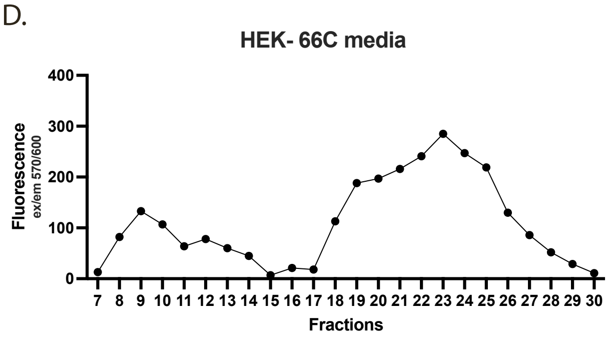


D) Fluorescent signal in different fractions derived via size exclusion chromatography from HEK cells transfected with the 66C construct. Related to Figure 2.

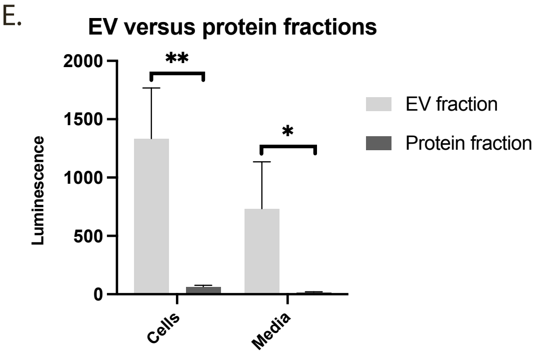


E) EVs and free proteins were isolated from N65 transfected HEK293T cells with size-exclusion chromatography and added to 66C transfected cells. The amount of EVs and protein added was normalized based on fluorescence. Nluc luminescence was then measured. Student's independent t-test, *: p<0.05, **: p<0.01. Related to Figure 2.
