# Supplementary material for: Illuminating cellular and extracellular vesicle-mediated communication via a split-Nanoluc reporter in vitro and in vivo
Source: Cell Rep Methods. 2023 Feb 21;3(2):100412. doi: 10.1016/j.crmeth.2023.100412 (PMC10014296; doi:10.1016/j.crmeth.2023.100412)
Supplement: Document S2. Article plus supplemental information [file mmc2.pdf]

Cell Reports
Methods

Report

Illuminating cellular and extracellular vesicle-mediated communication via a split-Nanoluc reporter *in vitro* and *in vivo*

Graphical abstract

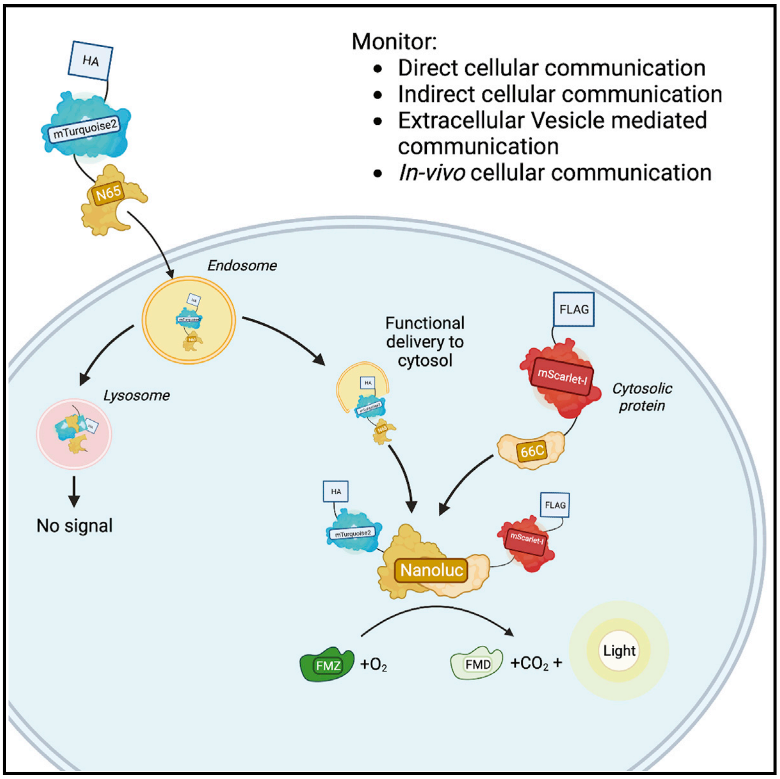

Highlights

- The split Nanoluc reporter system can be used to monitor functional protein uptake
- Direct, indirect, and extracellular vesicle (EV)-mediated communication can be detected
- The system also allows for monitoring exchange of proteins *in vivo*

Authors

Thomas S. van Solinge, Shadi Mahjoum, Stefano Ughetto, Alessandro Sammarco, Marike L.D. Broekman, Xandra O. Breakefield, Killian P. O'Brien

Correspondence

killianpob@gmail.com

In brief

van Solinge et al. develop a fluorescently labeled split Nanoluc reporter system to demonstrate and quantify functional transfer of proteins between cells *in vitro* and in a subcutaneous tumor mouse model. The construct allows monitoring of direct, indirect, and specifically extracellular vesicle mediated functional communication.

van Solinge et al., 2023, Cell Reports Methods *3*, 100412
February 27, 2023 © 2023 The Authors.
https://doi.org/10.1016/j.crmeth.2023.100412

CellPress

Cell Reports Methods

CellPress
OPEN ACCESS

Report

Illuminating cellular and extracellular vesicle-mediated communication via a split-Nanoluc reporter *in vitro* and *in vivo*

Thomas S. van Solinge,[1,2,7] Shadi Mahjoum,[1,7] Stefano Ughetto,[1,3] Alessandro Sammarco,[1,4,5] Marike L.D. Broekman,[1,2,6] Xandra O. Breakefield,[1] and Killian P. O'Brien[1,8,*]

[1]Molecular Neurogenetics Unit, Department of Neurology and Center for Molecular Imaging Research, Department of Radiology, Massachusetts General Hospital and Harvard Medical School, Boston, MA, USA
[2]Department of Neurosurgery, Leiden University Medical Center, Leiden, the Netherlands
[3]Department of Oncology, University of Turin, Candiolo, Italy
[4]Department of Microbiology, Immunology, and Molecular Genetics, University of California, Los Angeles, Los Angeles, CA, USA
[5]Department of Comparative Biomedicine and Food Science, University of Padua, Legnaro, Italy
[6]Department of Neurosurgery, Haaglanden Medical Center, The Hague, the Netherlands
[7]These authors contributed equally
[8]Lead contact
*Correspondence: killianpob@gmail.com
https://doi.org/10.1016/j.crmeth.2023.100412

MOTIVATION In tracking protein-mediated cellular communication, few tools distinguish between proteins that escape the endosome and are functional within the cytosol and proteins that are degraded by the lysosome or released back into the extracellular space. We designed a tool that signals functional protein delivery and can be used to study cell-to-cell and extracellular vesicle-mediated communication *in vitro* and *in vivo*.

SUMMARY

Tools to effectively demonstrate and quantify functional delivery in cellular communication have been lacking. This study reports the use of a fluorescently labeled split Nanoluc reporter system to demonstrate and quantify functional transfer between cells *in vitro* and in a subcutaneous tumor mouse model. Our construct allows monitoring of direct, indirect, and specifically extracellular vesicle-mediated functional communication.

INTRODUCTION

Intercellular communication is an integral part of maintaining homeostasis in multicellular organisms, with dysregulation playing pivotal roles in tumorigenesis,[1] aging,[2] and infectious disease.[3] Cellular communication is generally separated into direct, contact-dependent communication[4] and indirect communication, which is based on the exchange of soluble factors and extracellular vesicles.[5] Extracellular vesicles, membrane encapsulated particles released from cells via outward budding of the cellular plasma membrane or via fusion of endosomal-derived multivesicular bodies with the plasma membrane, have gathered special interest due to increasing evidence that their contents are selectively packaged and released, and their effects can be exerted far from the cell of origin.[6]

There are many tools to study the effects of cellular communication *in vitro* and *in vivo,* but those able to do so in real time are scarce.[7] Until now, much work has been limited to monitoring internalization as a means of evaluating functional uptake or analyzing indirect downstream targets.[7] Functional transfer of RNAs via extracellular vesicles (EVs) has been demonstrated through CRISPR-Cas9-based reporter systems, and these tools can be valuable on a single-cell level.[8] However, these are on-off systems where the readout does not equate to the amount of functionally delivered cargo, and translation into *in vivo* models is complex.[9] Quantification through luminescence is possible when using the commercially available reporter systems (Promega HI, Lgbit); however, these lack fluorescence,[10] making tracking of uptake difficult.

We set out to create a system that would allow for monitoring and quantification of functional protein exchange between cells for direct and indirect cell-to-cell interaction, including EV-mediated communication.

RESULTS

We created two proteins, N65 and 66C, which incorporate a previously developed split Nanoluc protein.[11] These two equivalently

This is an open access article under the CC BY license (http://creativecommons.org/licenses/by/4.0/).

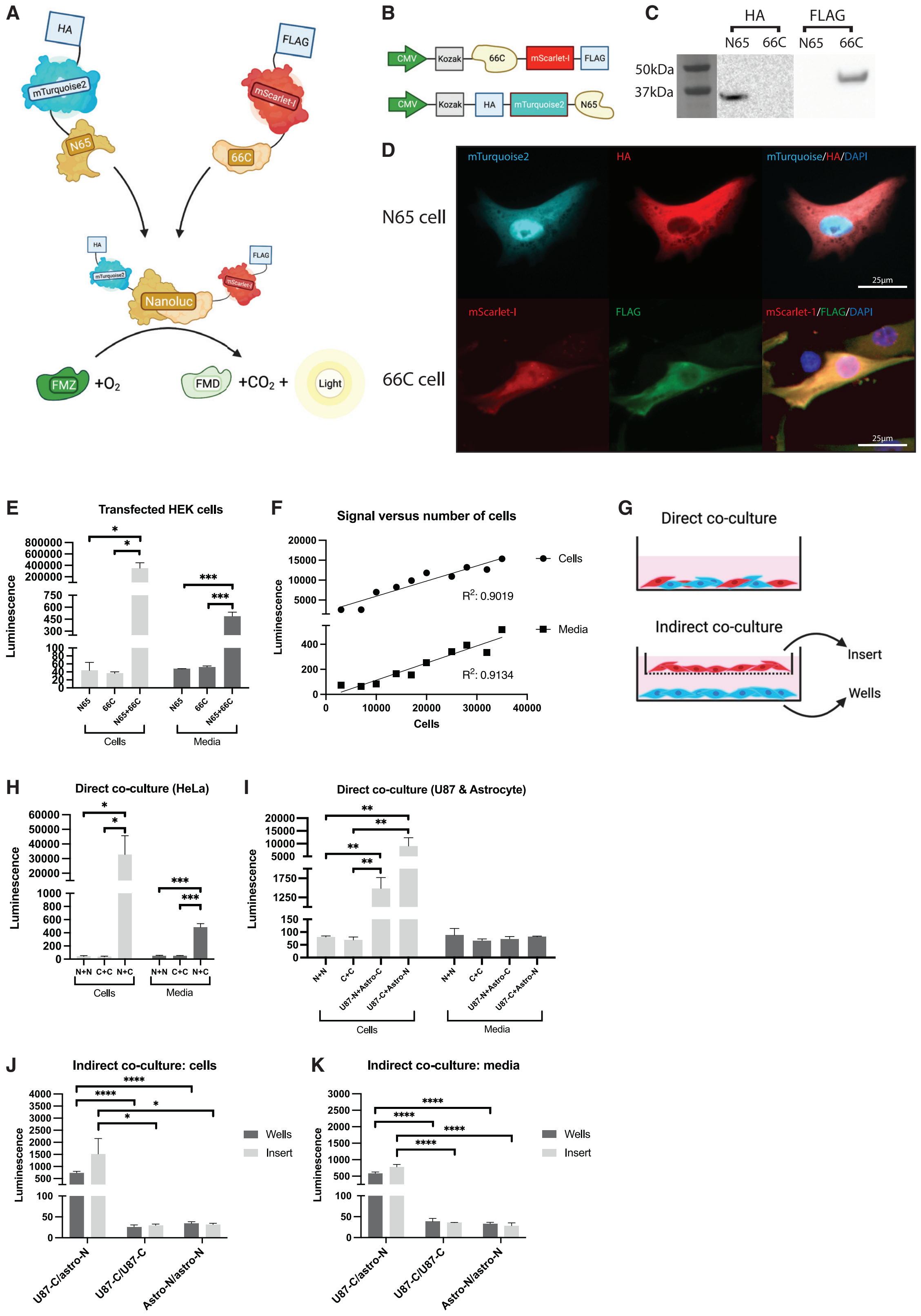

(legend on next page)

CellPress
OPEN ACCESS

sized fragments (N65 is 10.3 kDa and 66C is 13.5 kDa) spontaneously reform when in close proximity to one another. Nanoluc has no innate luminescence but catalyzes a bright stable luminescent signal when incubated with the substrate furimazine (FMZ).[12] We generated fusion proteins of these Nanoluc "halves": N65, with fluorescent protein mTurquoise-2[13] and a human influenza hemagglutinin (HA) tag[14] on the N terminus, and 66C, with mScarlet-I[15] and a FLAG tag[16] on the C terminus (Figures 1A–1D). Transfection of HEK cells with both constructs showed a strong luminescent signal in both the cells and the media when adding FMZ 48 h after transfection (Figure 1E). There was a strong correlation between the number of transfected cells and the luminescent signal in the cells and media, with $p < 0.0001$ and $R^2 > 0.9$ for both (Figure 1F). We evaluated functional delivery of proteins via direct and indirect culturing methods (Figure 1G). Direct co-culture of HeLa cells led to a strong luminescent signal in the cells and media after 7 days (Figure 1H). Similarly, we could detect luminescence in cells and media after direct co-culture of breast cancer cell line MDA-MB-231 (Figure S1A) and immortalized human astrocytes (Figure S1B). Functional exchange of proteins was also observed in direct co-culture of two different cell types: human-derived glioma cell line U87 and immortalized human astrocytes, although the signal could only be detected in the cells and not in the media (Figure 1I). The signal strength varied between experiments and was highly dependent on the number of cells fully transduced or the transfected level of transduction per cell as determined by fluorescent signal, total number of cells (as illustrated in Figure 1F), confluency, and length of co-culture (data not shown).

To assess if our tool was able to visualize indirect cellular communication, we performed indirect co-cultures with transduced U87 cells and immortalized astrocytes. We detected a luminescent signal in the cells and in the media (Figures 1J and 1K). This could indicate either free release and uptake of proteins or EV-mediated transfer of proteins. To evaluate the usefulness of our construct as an EV reporter, we transfected HEK cells with both constructs. Transfection agent was removed after 18 h, and cells were replated in fresh media. Media were collected after 72 h and run through a size-exclusion chromatographer (SEC). Most of the luminescent signal was observed in the protein fractions, while some signal could be observed in the early, EV-associated, fractions (7–11) (Figure S1C). Previously, we demonstrated that SEC successfully separates and isolates EVs from free proteins.[17] EV-specific protein CD81[18] could be detected in the EV fractions but not in the protein fractions (Figure S2A). To demonstrate that there was no contamination from free proteins in the EV fractions, we treated both EV and protein-associated fractions with proteinase K (Figure S2B). Proteinase K is a strong proteolytic enzyme that efficiently degrades proteins upon incubation.[19] EVs and proteins were collected from HEK293T cells transfected with both 66c and N65 constructs with SEC, and input was normalized based on fluorescence. The luminescent signal was similar between samples before adding proteinase K, while luminescence significantly decreased after adding proteinase K to the protein fractions. The signal in the EV fraction did not change, indicating that the proteins are indeed protected by the EV membrane and that there is little contamination of free proteins in the EV fractions (Figure S2B).

After stable transduction HeLa cells, mTurquoise2 and mScarlet-I fluorescence could be detected in the respective EV fractions (Figures 2A, 2B, S2C, and S2D). Next, concentrated EVs were added to HeLa cells transduced with the N65 or the 66C construct. Luminescence was measured after 48 h and could be detected in the recipient cells and in the media (Figures 2C and 2D). Repeated measurements from the moment of incubation with EVs and FMZ (t = 0), showed that the luminescence increased over time (Figures 2E and 2F). Finally, we compared the luminescent signal between delivery via EVs and delivery via free proteins. EVs and proteins were isolated from transfected N65 cells via SE, and the concentration of N65 protein was normalized based on fluorescence. Equal amounts were then added to transduced 66C recipient cells. Luminescence was measured after 72 h. With similar protein input, luminescent signal above baseline could be detected in 66C cells receiving N65 EVs and N65 free protein and in the media of cells that received N65 EVs (Figure S2E). A higher luminescent signal was detected when EVs were added compared with free protein.

To further demonstrate versatility of this tool, we fused a nuclear localization signal (NLS)[20] to the N65 construct (Figure 2G). The NLS causes accumulation of the protein in the cell nucleus, decreasing release into the cytosol and extracellular

Figure 1. Validation of the split Nanoluc construct and functional delivery

(A) Schematic illustration of the mechanism. The split halves of the Nanoluc fuse when in proximity to one another. Nanoluc then oxidizes furimazine (FMZ) to furimamide (FMD), carbon dioxide (CO_2), and light. HA, human influenza hemagglutinin.

(B) Design of the construct. CMV, cytomegalo virus.

(C) Western blot of HEK cell lysates stained with anti-FLAG and anti-HA. kDa, kilodalton.

(D) Fluorescent imaging of N65 and 66C transduced HeLa cells. DAPI, 4′,6-diamidino-2-phenylindole. Scale bar: 25 μm.

(E) HEK cells transfected with either one or both constructs. Signal measured in media and in cell lysate after adding FMZ for 1 min (n = 3 per condition, SEM). Student's independent t test. *$p < 0.05$, ***$p < 0.001$.

(F) Correlation between luminescence and cell number in transfected HEK cells, as measured in cell lysate and media. One replicate per condition. Linear regression analysis, $p < 0.0001$ for both.

(G) Schematic of direct and indirect co-culture of cells.

(H) Direct co-culture of transduced HeLa cells. N65 (N) or 66C (C) was cultured. Luminescence could be detected in the cell lysate and media after adding FMZ. Student's independent t test. *$p < 0.05$, ***$p < 0.001$ (n = 3 per condition, SEM).

(I) Direct co-culture of U87 cells and astrocytes transduced with either N65 (N) or 66C (C). Strong signal was detected in the cell lysate but not media. Student's independent t test. **$p < 0.01$ (n = 3 per condition, SEM).

(J) Indirect co-culture of U87-66C and astrocyte-N65 cells. Luminescent signal in cells. Student's independent t test. *$p < 0.05$, ****$p < 0.0001$ (n = 3 per condition, SEM).

(K) Indirect co-culture of U87-66C and astrocyte-N65 cells. Luminescent signal in media. Student's independent t test. ****$p < 0.0001$ (n = 3 per condition, SEM).

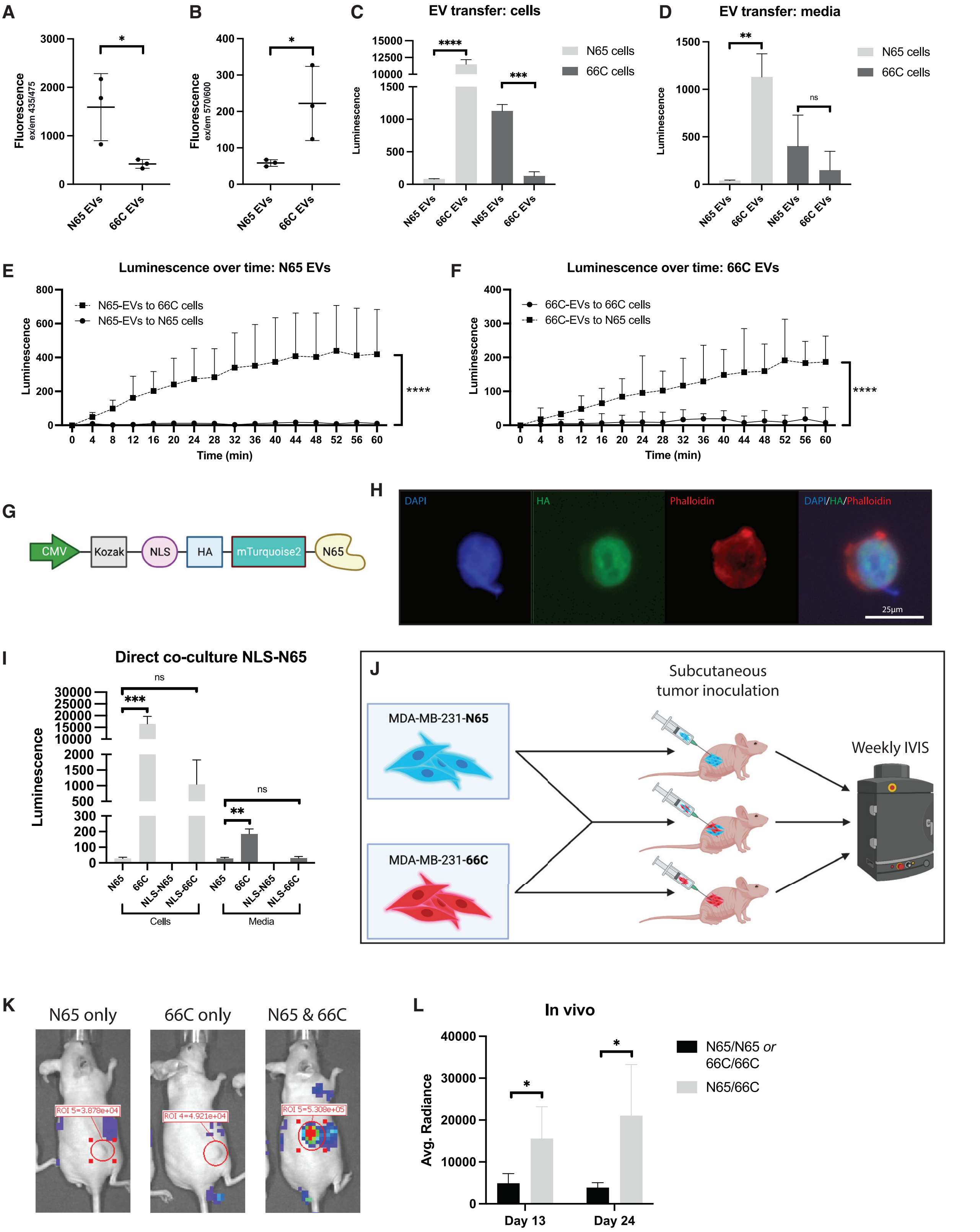

(legend on next page)

CellPress
OPEN ACCESS

compartment (Figure 2H). Co-culturing of NLS-N65 transduced HEK cells demonstrated retention of the NLS-fused protein in the nucleus, prohibiting functional uptake in the other cells (Figure 2I). Luminescence was measured in the cell pellet and media 7 days after the start of co-culture. As expected, there was no significant signal in cells and media when co-culturing NLS-N65 with N65 or NLS-N65 HEK cells. A significant increase in luminescence was found when co-culturing NLS-N65 with normal 66C HEK cells but not when co-cultured with NLS-66C. When both N65 and 66C proteins are fused with NLS, they do not leave the nucleus, and thus functional transfer to other cells is inhibited. In this experiment, some increase in luminescence was observed when co-culturing NLS-N65 and NLS-66C compared with control, indicating some leakage of proteins out of the nucleus, but this increase was not significant and not observed in the media (Figure 2I). Direct co-culture of two NLS constructs did not lead to significant increase in luminescent signal, indicating successful retention of the protein to the nucleus (Figure 2I).

Finally, cellular communication could be detected *in vivo*. Breast cancer cells MDA-MB-231 were transduced with either the N65 construct or the 66C construct and grown for 7 days. Athymic nude mice were subcutaneously injected with MDA-MB-231-N65 or MDA-MB-231-66C cells or with a mixture of both cell lines. Rapid tumor formation ensued. To visualize functional exchange of proteins, we injected fluorofurimazine (FFz), optimized for *in vivo* applications of Nanoluc,[21] intraperitoneally in mice weekly and measured *in vivo* bioluminescence (Figure 2J). We were able to detect a strong luminescent signal in the tumors consisting of both cell lines compared with controls with only one cell line, which increased over time (Figures 2K and 2L). This indicates that this construct can be utilized to study cellular communication *in vivo.*

DISCUSSION

In this report, we demonstrate an assay to measure functional delivery of proteins for direct, indirect, and EV-mediated communication. We show the functionality and adaptability of these assays *in vitro* and *in vivo.*

Many assays have been developed to measure (EV-mediated) delivery, all with various qualities and shortcomings. Recently a model utilizing the LgBit and HiBit tags was developed, following a similar concept to show functional delivery of proteins.[22] Specifically designed to demonstrate functional delivery via EVs, delivery could be detected via luminescence in real time. As LgBit and HiBit weakly associate, the efficacy of Nanoluc formation depends on the interaction of the target proteins to which they are fused.[12] The rate of Nanoluc formation was too low to be detected, and only after addition of a fusogenic protein could the signal be detected above background.[22] Toribio et al. attempted to create a similar assay using a split EGFP luciferase, with one-half fused to CD9 and the other half freely expressed within recipient cells, but this did not provide a fluorescent or luminescent signal upon EV-mediated delivery.[23] Only when EVs carried the full reconstituted dual-EGFP-Renilla protein and the cyto-permeable Renilla luciferase substrate could uptake be detected. While it can show live uptake and is quantifiable, this assay is not able to distinguish between uptake and actual functional delivery.[23] Similarly, another study was able detect GFP fused to CD63 in donor cells incubated with GFP-CD63 EVs, showing uptake but not specifying functional delivery.[24] Functional delivery of RNAs has been demonstrated by a study utilizing the CRISPR-Cas9 system.[8] Cells were transduced to express mCherry, with EGFP flanked by a stop codon. Upon functional delivery of a single guide RNA, CRISPR-Cas9 removes one or two nucleotides in the linker, creating a frameshift to bypass the stop codons and transcribe the EGFP. Functionality was shown in co-culture and EV-based assays, which demonstrated low efficiency: on average, 0.07% of cells expressed GFP after 5 days of incubation.[8]

Our construct offers various advantages over currently available protein-based assays. First, we demonstrate the versatility of this construct, showing its use in direct, indirect, and EV-based cellular communication. As two intact proteins are

Figure 2. Functional delivery in EVs, modification of the construct, and *in vivo* possibilities
(A) Fluorescence of EVs isolated from transfected HEK cells via size exclusion (SE) excited at 435 nm. Student's independent t test. *$p < 0.05$ ($n = 3$ per condition, SEM).
(B) Fluorescence of EVs excited at 570 nm. Student's independent t test. *$p < 0.05$ ($n = 3$ per condition, SEM).
(C) EVs isolated from transfected HEK cells and added to transduced HEK-N65 and HEK-66C cells. Luminescence in cells after addition of FMZ. Student's independent t test. ***$p < 0.001$, ****$p < 0.0001$ ($n = 3$ per condition, SEM).
(D) EVs isolated from transfected HEK cells and added to transduced HEK-N65 and HEK-66C cells. Luminescence in media after addition of FMZ. Student's independent t test. ns, not significant. **$p < 0.01$ ($n = 3$ per condition, SEM).
(E) Luminescence over time upon adding HEK-N65-derived EVs. EVs and FMZ were added at $t = 0$. Difference between HEK-66c and HEK-N65 cells at $t = 60$ min, independent Student's t test: ****$p < 0.0001$ ($n = 3$ per condition, SEM).
(F) Luminescence over time upon adding HEK-66C-derived EVs. EVs and FMZ were added at $t = 0$. Difference between HEK-66c and HEK-N65 cells at $t = 60$ min, Student's t test: ****$p < 0.0001$ ($n = 3$ per condition, SEM).
(G) Nuclear-localizing construct for N65 cells. A nuclear-localizing signal (NLS) was added to the HA-mTurquoise2-N65 construct, fusing it to the split Nanoluc.
(H) HEK cells transduced with the NLS-N65 construct. The signal is localized to the nucleus, with no fluorescence seen in the cell membrane, as visualized with phalloidin staining. 25 μm.
(I) Direct co-culture of NLS and normal constructs. When the protein is contained in the nucleus, a decrease in signal is seen. Student's independent t test. ns, not significant. **$p < 0.01$, ***$p < 0.001$ ($n = 3$ per condition, SEM).
(J) Setup for the *in vivo* experiment. IVIS, *in vivo* imaging system.
(K) Examples of nude mice injected with MDA-MB-231-N65, MDA-MB-231-66c, or both tumor lines. Measurement of luminescence 10 min after injection of fluorofurimazine.
(L) Average radiance 10 min after injection of fluorofurimazine on days 13 and 24 of tumor injection. Student's independent t test. *$p < 0.05$ ($n = 4$ per condition, SEM).

required to create the luminescent protein, this assay distinguishes uptake from functional delivery. Furthermore, we show that this construct works both ways, with both halves being suitable to be either donor or receiver, and that the construct can be adapted to study specific aspects of cellular communication without a significant decrease in functionality. This construct uniquely allows monitoring of cellular communication in an *in vivo* mouse model, with the signal being strong enough to be detected with IVIS.

Overall, our reporter system provides a tool that allows for detection and relative quantification of direct, indirect, and specifically indirect EV-mediated cellular interaction.

We believe that this tool will facilitate researchers to evaluate and improve cellular communication and functional EV-mediated delivery and further aid our understanding of cellular communication *in vitro* and *in vivo.*

Limitations of the study

One of the limits of this construct is the difficulty in quantifying the signal. Strength of signal depends on many variables, such as strength of transduction or transfection, number of cells, EVs released, amount of protein packed per EV, and many others. Another limitation is that the construct does not differentiate between methods of delivery; the luminescent signal demonstrates that functional delivery has occurred, not whether this has been through proteins, EVs, or other forms of cellular communication. By carefully controlling experiments, this construct can be used to assess individual forms of cellular communication, but contamination cannot be ruled out based on this construct alone. Furthermore, luminescence does tend to vary between experiments. Comparing functional uptake between two different experiments is therefore difficult, and caution needs to be taken when extrapolating results from single experiment. While other constructs have similar issues, the CRISPR-Cas9 method does allow for better quantifiable response.[8] Functionality of the Nanoluc is, however, not dependent on post-translational modification, as are GFP-based reporter systems,[25] allowing for it to function at lower expression levels with less influence of other cellular processes.[26]

STAR★METHODS

Detailed methods are provided in the online version of this paper and include the following:

- KEY RESOURCES TABLE
- RESOURCE AVAILABILITY
  - Lead contact
  - Materials availability
  - Data and code availability
- EXPERIMENTAL MODEL AND SUBJECT DETAILS
  - Cell lines
  - Animal studies
- METHOD DETAILS
  - Cloning
  - Western Blot
  - Transfection
  - Lentiviral production
  - Cell transduction
  - EV isolation
  - Luminescent assay
  - Proteinase K treatment
  - EV transfer experiment
  - Direct Co-Culture
  - Indirect Co-Culture
  - Immunocytochemistry
  - Bioluminescence imaging in mice
- QUANTIFICATION AND STATISTICAL ANALYSIS

SUPPLEMENTAL INFORMATION

Supplemental information can be found online at https://doi.org/10.1016/j.crmeth.2023.100412.

ACKNOWLEDGMENTS

A.S. is supported by an American-Italian Cancer Foundation Postdoctoral Research Fellowship. T.S.v.S., S.M., X.O.B., and K.P.O'B. are supported by NIH grant P01 CA069246. T.S.v.S., S.M., X.O.B., K.P.O'B., and M.B. are supported by grant NIH NCI R35 CA232103. T.S.v.S. is supported by grants from the Bontius Stichting, the Nijbakker-Morra Fund, Foundation Vrijvrouwe van Renswoude, and the Bekker-la Bastide Fund.

AUTHOR CONTRIBUTIONS

Conceptualization, K.P.O'B. and X.O.B.; methodology, K.P.O'B., X.O.B., T.S.v.S., and S.M.; validation, T.S.v.S., S.M., S.U., and A.S.; formal analysis, T.S.v.S., S.M., and K.P.O'B.; investigation, K.P.O'B., T.S.v.S., S.M., S.U., and A.S.; resources, X.O.B.; writing – original draft, T.S.v.S. and S.M.; writing – review & editing, T.S.v.S., S.M., S.U., A.S., M.B., X.O.B., and K.P.O'B.; visualization, T.S.v.S. and S.M.; supervision, K.P.O'B., X.O.B., and M.B.; project administration, K.P.O'B. and X.O.B.; funding acquisition, X.O.B. and M.B.

DECLARATION OF INTERESTS

The authors declare no competing interests.

INCLUSION AND DIVERSITY

One or more of the authors of this paper self-identifies as an underrepresented ethnic minority in their field of research or within their geographical location. We support inclusive, diverse, and equitable conduct of research.

Received: March 30, 2022
Revised: September 1, 2022
Accepted: January 30, 2023
Published: February 21, 2023

REFERENCES

1. Brücher, B.L.D.M., Jamall, I.S., and Jamall, I.S. (2014). Cell-cell communication in the tumor microenvironment, carcinogenesis, and anticancer treatment. Cell. Physiol. Biochem. *34*, 213–243. https://doi.org/10.1159/000362978.

2. Fafián-Labora, J.A., and O'Loghlen, A. (2020). Classical and nonclassical intercellular communication in senescence and ageing. Trends Cell Biol. *30*, 628–639. https://doi.org/10.1016/J.TCB.2020.05.003.

3. Alto, N.M., and Orth, K. (2012). Subversion of cell signaling by pathogens. Cold Spring Harb. Perspect. Biol. *4*, a006114. https://doi.org/10.1101/CSHPERSPECT.A006114.

CellPress
OPEN ACCESS

4. Mattes, B., and Scholpp, S. (2018). Emerging role of contact-mediated cell communication in tissue development and diseases. Histochem. Cell Biol. *150*, 431–442. https://doi.org/10.1007/S00418-018-1732-3.

5. Abels, E.R., and Breakefield, X.O. (2016). Introduction to extracellular vesicles: biogenesis, RNA cargo selection, content, release, and uptake. Cell. Mol. Neurobiol. *36*, 301–312. https://doi.org/10.1007/s10571-016-0366-z.

6. Maas, S.L.N., Breakefield, X.O., and Weaver, A.M. (2017). Extracellular vesicles: unique intercellular delivery vehicles. Trends Cell Biol. *27*, 172–188. https://doi.org/10.1016/j.tcb.2016.11.003.

7. Yang, B.A., Westerhof, T.M., Sabin, K., Merajver, S.D., and Aguilar, C.A. (2021). Engineered tools to study intercellular communication. Adv. Sci. *8*, 2002825. https://doi.org/10.1002/ADVS.202002825.

8. de Jong, O.G., Murphy, D.E., Mäger, I., Willms, E., Garcia-Guerra, A., Gitz-Francois, J.J., Lefferts, J., Gupta, D., Steenbeek, S.C., van Rheenen, J., et al. (2020). A CRISPR-Cas9-based reporter system for single-cell detection of extracellular vesicle-mediated functional transfer of RNA. Nat. Commun. *11*, 1113. https://doi.org/10.1038/s41467-020-14977-8.

9. Wilbie, D., Walther, J., and Mastrobattista, E. (2019). Delivery aspects of CRISPR/Cas for in vivo genome editing. Acc. Chem. Res. *52*, 1555–1564. https://doi.org/10.1021/ACS.ACCOUNTS.9B00106.

10. Boursier, M.E., Levin, S., Zimmerman, K., MacHleidt, T., Hurst, R., Butler, B.L., Eggers, C.T., Kirkland, T.A., Wood, K.V., and Friedman Ohana, R. (2020). The luminescent HiBiT peptide enables selective quantitation of G protein-coupled receptor ligand engagement and internalization in living cells. J. Biol. Chem. *295*, 5124–5135. https://doi.org/10.1074/JBC.RA119.011952.

11. Zhao, J., Nelson, T.J., Vu, Q., Truong, T., and Stains, C.I. (2016). Self-assembling NanoLuc luciferase fragments as probes for protein aggregation in living cells. ACS Chem. Biol. *11*, 132–138. https://doi.org/10.1021/acschembio.5b00758.

12. Dixon, A.S., Schwinn, M.K., Hall, M.P., Zimmerman, K., Otto, P., Lubben, T.H., Butler, B.L., Binkowski, B.F., MacHleidt, T., Kirkland, T.A., et al. (2016). NanoLuc complementation reporter optimized for accurate measurement of protein interactions in cells. ACS Chem. Biol. *11*, 400–408. https://doi.org/10.1021/acschembio.5b00753.

13. Goedhart, J., Von Stetten, D., Noirclerc-Savoye, M., Lelimousin, M., Joosen, L., Hink, M.A., Van Weeren, L., Gadella, T.W.J., and Royant, A. (2012). Structure-guided evolution of cyan fluorescent proteins towards a quantum yield of 93%. Nat. Commun. *3*, 751. https://doi.org/10.1038/ncomms1738.

14. Tai, T.N., Havelka, W.A., and Kaplan, S. (1988). A broad-host-range vector system for cloning and translational lacZ fusion analysis. Plasmid *19*, 175–188. https://doi.org/10.1016/0147-619X(88)90037-6.

15. Bindels, D.S., Haarbosch, L., Van Weeren, L., Postma, M., Wiese, K.E., Mastop, M., Aumonier, S., Gotthard, G., Royant, A., Hink, M.A., and Gadella, T.W.J., Jr. (2017). MScarlet: a bright monomeric red fluorescent protein for cellular imaging. Nat. Methods *14*, 53–56. https://doi.org/10.1038/nmeth.4074.

16. Terpe, K. (2003). Overview of tag protein fusions: from molecular and biochemical fundamentals to commercial systems. Appl. Microbiol. Biotechnol. *60*, 523–533. https://doi.org/10.1007/s00253-002-1158-6.

17. O'Brien, K., Ughetto, S., Mahjoum, S., Nair, A.V., and Breakefield, X.O. (2022). Uptake, functionality, and re-release of extracellular vesicle-encapsulated cargo. Cell Rep. *39*, 110651. https://doi.org/10.1016/j.celrep.2022.110651.

18. Yoshioka, Y., Konishi, Y., Kosaka, N., Katsuda, T., Kato, T., and Ochiya, T. (2013). Comparative marker analysis of extracellular vesicles in different human cancer types. J. Extracell. Vesicles *2*, 20424. https://doi.org/10.3402/jev.v2i0.20424.

19. EBELING, W., HENNRICH, N., KLOCKOW, M., METZ, H., ORTH, H.D., and LANG, H. (1974). Proteinase K from tritirachium album limber. Eur. J. Biochem. *47*, 91–97. https://doi.org/10.1111/J.1432-1033.1974.TB03671.X.

20. Dos Reis Figueira, A., Golem, S., Goregaoker, S.P., and Culver, J.N. (2002). A nuclear localization signal and a membrane association domain contribute to the cellular localization of the Tobacco mosaic virus 126-kDa replicase protein. Virology *301*, 81–89. https://doi.org/10.1006/viro.2002.1560.

21. Su, Y., Walker, J.R., Park, Y., Smith, T.P., Liu, L.X., Hall, M.P., Labanieh, L., Hurst, R., Wang, D.C., Encell, L.P., et al. (2020). Novel NanoLuc substrates enable bright two-population bioluminescence imaging in animals. Nat. Methods *17*, 852–860. https://doi.org/10.1038/s41592-020-0889-6.

22. Somiya, M., and Kuroda, S. (2021). Real-time luminescence assay for cytoplasmic cargo delivery of extracellular vesicles. Anal. Chem. *93*, 5612–5620. https://doi.org/10.1021/acs.analchem.1c00339.

23. Toribio, V., Morales, S., López-Martín, S., Cardeñes, B., Cabañas, C., and Yáñez-Mó, M. (2019). Development of a quantitative method to measure EV uptake. Sci. Rep. *9*, 10522. https://doi.org/10.1038/s41598-019-47023-9.

24. Joshi, B.S., de Beer, M.A., Giepmans, B.N.G., and Zuhorn, I.S. (2020). Endocytosis of extracellular vesicles and release of their cargo from endosomes. ACS Nano *14*, 4444–4455. https://doi.org/10.1021/acsnano.9b10033.

25. Coralli, C., Cemazar, M., Kanthou, C., Tozer, G.M., and Dachs, G.U. (2001). Limitations of the reporter green fluorescent protein under simulated tumor conditions. Cancer Res. *61*, 4784–4790.

26. England, C.G., Ehlerding, E.B., and Cai, W. (2016). NanoLuc: a small luciferase is brightening up the field of bioluminescence. Bioconjug. Chem. *27*, 1175–1187. https://doi.org/10.1021/acs.bioconjchem.6b00112.

STAR★METHODS

KEY RESOURCES TABLE

REAGENT or RESOURCE	SOURCE	IDENTIFIER
Antibodies		
Anti-HA tag, mouse	AbCam	Cat: 18181 RRID:AB_444303
Anti-FLAG, mouse	Sigma-Aldrich	Cat: F3165 RRID:AB_259529
Goat anti-mouse IgG, HRP	Thermo Fisher	Cat: 62-6520 RRID:AB_2533947
Goat anti-mouse IgG, Alexa Fluor 546	Thermo Fisher	Cat: A-11030 RRID:AB_2534089
Goat anti-mouse IgG, Alexa Fluor 488	Thermo Fisher	Cat: A-32723 RRID:AB_2633275
Critical commercial assays		
Nano-Glo luciferase	Promega	Cat: N1120
Nano-Glo *In Vivo* Substrate	Promega	On request from manufacturer
Experimental models: Cell lines		
Human embryonic kidney 293 (HEK293T)	ATCC	CRL-3216; RRID:CVCL_0063
HeLa Cells	ATCC	Cat: 300194/p772_HeLa; RRID:CVCL_0030
U87	ATCC	Cat: HTB-14(tm) RRID:CVCL_0022
MDA-MB-231	ATCC	Cat: HTB-26(tm) RRID:CVCL_0062
Human astrocytes	ScienCell	Cat: 1800 RRID:CVCL_B5WG
Experimental models: Organisms/strains		
Athymic nude mice (nu/nu)	Charles River laboratories	Cat: 553NCIATH/NU
Recombinant DNA		
psPAX2	Addgene	Cat: 12260
pMD2.G	Addgene	Cat: 12259
Software and algorithms		
Adobe Illustrator	Adobe	Version 24.3
Prism	Graphpad	Version 9.4.1
Microsoft Excel	Microsoft	Version 16.6
Biorender	Biorender.com	N/A

RESOURCE AVAILABILITY

Lead contact

Further information and requests for resources and reagents should be directed to and will be fulfilled by the lead contact, Killian O'Brien (killianpob@gmail.com).

Materials availability

This study did not generate new unique reagents.

Data and code availability

- All data generated and analyzed in this study are available from the corresponding author on reasonable request.
- This paper does not report original code.
- Any additional information required to reanalyze the data reported in this paper is available from the lead contact upon request.

EXPERIMENTAL MODEL AND SUBJECT DETAILS

Cell lines

Human embryonic kidney 293 (HEK293T), HeLa, U-87, and MDA-MB-231 cells, all from American Type Culture Collection (ATCC; Manassas, VA, USA) were cultured at 37°C in a 5% CO2 humidified incubator. Culture media for HEK293T, HeLa, U-87, and

MDA-MB-231 cells was Dulbecco's Modified Essential Medium (DMEM; Corning) with L-glutamine (Corning) supplemented with penicillin (100 units/mL), streptomycin (100 mg/mL) (P/S) (Corning) and 10% fetal bovine serum (FBS; Atlanta Biologics). Culture media for astrocytes consisted of basal medium, 2% FBS, (Cat. No. 0010), 5 mL of astrocyte growth supplement (AGS, Cat. No. 1852) and 5 mL of penicillin/streptomycin solution (P/S, Cat. No. 0503). Stable fluorescent cell lines were generated by lentiviral transduction with N65 and 66C constructs. Cells were routinely tested for mycoplasma contamination (Mycoplasma PCR Detection Kit, abm G238) and found negative.

Animal studies

Ten week old, female athymic nude mice (nu/nu) were obtained from Charles River Laboratories. Per mouse, 1×10^6 cells (MDA-MB-231-66C only, MDA-MB-231-N65 only, or MDA-MB-231-66C and MDA-MB-231-N65) were suspended in 50 μL 0.9% sodium chloride (Hospira, Lake Forest, IL, USA) and mixed with 50 μl Matrigel Matrix (10 mg/mL. Corning, NY, USA.). Mice were anesthetized with isoflurane and placed on a heating pad to maintain body temperature. The cells were injected subcutaneously in the lower right flank. Tumor growth was monitored biweekly. Mice were euthanized when tumors reached 500 mm^3 in volume, or if the tumor interfered with eating, drinking, defecating, or urinating, or if the tumor showed signs of ulcerating. All experiments were approved under IACUC protocol 2009N000054 by the Massachusetts General Hospital Center for Comparative Medicine.

METHOD DETAILS

Cloning

Sequences for N65 and 66C were taken from Zhao et al.[11] Sequences for mTurquoise2 and mScarlet-I were added to both, respectively, and ordered from Integrated DNA Technologies (IDT) as a gBlock and cloned into lentiviral backbone (Supplemental Information). We also added c-myc NLS sequence to localize expression to the nucleus, which was also ordered as a gBlock (Supplemental Information). Cloning was performed using Gibson Assembly Cloning kit from New England Biolabs (NEB). Plasmids were transformed in One ShotTOP10 bacterial cells from ThermoFisher Scientific. Subsequent MaxiPrep kits (ThermoFisher Scientific) were used to isolate DNA and samples were then sequenced by the MGH sequencing core.

Western Blot

Cells were trypsinized and lysed with RIPA buffer (Abcam, Cambridge, MA, USA) with a cocktail of Protease Inhibitors (Roche, Mannheim, Germany) and centrifuged for 10 min at 12,000 × g at 4°C. Protein concentration was quantified with the Pierce BCA Protein Assay kit (Thermo Scientific). 2μg of protein was denatured at 95°C for 5 min. Samples were run on 4–12% NuPAGE Bis-Tris Gel (ThermoFisher Scientific) and transferred to a nitrocellulose membrane (Bio-Rad). After blocking with Tris-Buffered Saline (TBS) with 0.05% Tween 20 (TBS-T) and 5% milk for 1 h, the membrane was incubated with the primary antibody in TBS-T with 3% milk overnight at 4°C. The membrane was washed with TBS-T and incubated with the secondary antibody for 1 h at room temperature. Primary antibodies were: Anti-HA tag, (mouse mAB, 18181, abcam), Anti-Flag (mouse mAB, F3165, Sigma-Aldrich), all 1:100 dilutions. Secondary was ECL Anti-mouse IgG (Thermo Fisher) (1:1000).

Transfection

To introduce plasmids into HEK293T cells, 1 μg/mL polyethylenimine (PEI) (Polyscience, Warrington, PA USA) was added to the media. DNA (4 μg) was mixed with 250 μL Opti-MEM® (ThermoFisher), and 50 μL PEI was mixed with 200 μL Opti-MEM separately. They were each incubated at RT for 5 min and then combined to a final volume of 500 μL. After the transfection agent was incubated at RT for 20 min, it was dropwise added to the cells and incubated for 18 h after which cells were trypsinized and washed with PBS before replating in fresh media.

Lentiviral production

Lentiviral production and transduction of cells was performed as described previously.

Lentiviral vectors encoding for N65, 66C, and NLS-N65 constructs were produced in HEK293T cells with a three-plasmid system, following Addgene recommendations. 10ˆ6 seeded cells were transfected with psPAX2 (#12260) and pMD2.G (#12259) packaging plasmids and the transgene of interest flanked by long terminal repeats (LTRs). Six hours after transfection, cells were washed with PBS, and fresh media was added. After 72 h, the media was collected, and viral isolation was performed by ultracentrifugation at 70,000 × g. The pellet was resuspended in 1% BSA in PBS. The viral particle content was evaluated by assessing HIV-1 p24 antigen levels by ELISA (Retro Tek, Gentaur, Paris, France). Concentrated viral stocks were then stored at −80°C until use.

Cell transduction

Cell lines were incubated in FBS-free media with lentiviral vectors for 72 h at different concentrations. Medium was subsequently replaced with new DMEM media and antibiotics. The stable transduced cell lines were cultured and expanded under conditions described above.

EV isolation

HEK293T cells were seeded in four 150 mm dishes (seeding density $\approx 2.5 \times 10^6$ cells) and grown for 48 h. The cells were transfected with the plasmids of interest and incubated for 18 h, after which new DMEM media was added. After 72 h, the media was collected from the cells and concentrated using UFC9100 Amicon® Ultra-15 Centrifugal filters (100 kDa), centrifuged at 6,000 × g for 15 min at 4°C until all media was concentrated. The concentrated sample (~500 µL) was added onto Izon qEVoriginal/70 nm columns installed in a size exclusion chromatographer (SEC). The Izon column was washed with 10 mL 1X phosphate buffer saline, pH 7.4 (PBS; Boston Bioproducts) between each sample. Fifteen mL PBS was added after the concentrated media had entered the Izon column to collect the fraction, using the Izon automatic fraction collector (AFC). Fractions 7 to 30 were collected for full profile analysis. For EV isolation, fraction 7 to 11 were concentrated using Amicon®Ultra-0.5 Centrifugal (30 kDa) centrifuged at 12,000 × g for 3 min. Transfer of each fraction to the Amicon filter was performed in a sterile manner (i.e., in fume hoods with UV irradiated filters).

Luminescent assay

To analyze NanoLuc luciferase (Nluc) expression, furimazine (Nano-Glo® Luciferase, Promega) was diluted to 1:500 in 1x PBS. The samples were loaded onto 96- well white bottom Greiner Bio-one plates and incubated with the reagent for at least 3 min prior to reading on the BioTek luminometer (Synergy H1 Hybrid Multi-Mode Reader). For the live uptake assay (Figures 2E and 2F), cells were washed with PBS and EVs concentrated in 30µL of PBS were added to the wells. One µL of furimazine was then directly added in each well, and the luminescence recorded every 4 min.

Proteinase K treatment

HEK293T cells were co-transfected with N65 and 66C constructs. EV fractions were collected after 72 h as described previously. Proteins were collected and up-concentrated with centrifugation through similar methods from fraction 14 and 15. Samples were then treated with 100ug/mL of Proteinase K (Thermo Fisher, cat: EO0491) or PBS for 8 min at 56°C, and immediately cooled on ice afterward. Sample were then analyzed for Nluc expression.

EV transfer experiment

Stably transduced cell lines were seeded in 96-well culture plates (seeding density 1×10^4 cells/well). EVs were added to the recipient cells 5 h after seeding. The cells were incubated with EVs for a maximum of 48 h. The media and cells were analyzed for Nluc expression.

Direct Co-Culture

HeLa N65 and HeLa 66C cell lines were seeded together in 24-well plates (seeding density 5×10^4 cells/well). On day 7, the cells were resuspended with 0.25% trypsin-EDTA (Thermofisher) for 5 min at 37°C. The cells were centrifuged at 300 × g for 5 min. The cell pellets were suspended in 100 µL 1x PBS and loaded onto 96-well white bottom Greiner Bio-one plates, FMZ diluted 1:500 was added, and plates were analyzed for luminescence.

Indirect Co-Culture

Stable transduced cell lines were seeded at 1.5×10^4 cells onto each insert (Transparent PET Membrane 6-well 1µm pores) and 3×10^4 cells in the wells of 6-well cell culture plates. Seven days after the cells were seeded, the medium and cells on the insert and in the bottom chamber were measured for luminescence as described above.

Immunocytochemistry

Cells were seeded and incubated on circular glass microscope coverslips in 24 well-plates for 24 h. The media was aspirated from the coverslips, and cells were washed with PBS three times and fixed in 4% paraformaldehyde (PFA; Electron Microscopy Sciences) for 20 min at RT. The cells were washed with PBS three times, 5 min each. The cells were blocked and permeabilized in 5% Bovine Serum Albumin (BSA) in PBS with 0.1% Triton-100 for 1 h at RT. The samples were incubated overnight at 4°C with primary antibodies 1:200 in PBS: Anti-HA tag, (mouse mAB, 18,181, abcam), Anti-Flag (mouse mAB, F3165, Sigma-Aldrich). The samples were washed with PBS three times, and incubated for 1 h at RT with secondary antibody diluted 1:500 in PBS: Alexa Fluor 546 (A-11030, Thermo-Fisher, Goat anti-mouse), Alexa Fluor 488, (A-32723, Thermo-Fisher, Goat anti-mouse). Samples were mounted in Vectashield® Antifade (Vector Laboratories) for imaging. Imaging was performed on BZ-X microscope (Keyence, Itasca, IL, United States). Analysis and post-production of images was done with ImageJ v2.0 (National Institute of Health, United States) and Adobe Illustrator (Adobe Inc. San Jose, CA, USA).

Bioluminescence imaging in mice

50 µL Fluorofurimazine (Nano-Glo *In Vivo* Substrate. Promega, Madison, WI, USA.) was injected intra-peritoneally in isoflurane anesthetized mice. After 10 min, mice were imaged using the IVIS (*In Vivo* Imaging System) spectrum (PerkinElmer, Waltham, MA, USA). Bioluminescence was compared at Average Radiance (protons/second/cm/steradian).

CellPress
OPEN ACCESS

QUANTIFICATION AND STATISTICAL ANALYSIS

Data were analyzed with Graphpad Prism, version 9.4.1 and Microsoft Excel, version 16.6. All statistical data can be found in figure legends with the number of replicaties, statistical test performed and p-values.

Cell Reports Methods, Volume *3*

Supplemental information

Illuminating cellular and extracellular vesicle-mediated communication via a split-Nanoluc reporter *in vitro* and *in vivo*

Thomas S. van Solinge, Shadi Mahjoum, Stefano Ughetto, Alessandro Sammarco, Marike L.D. Broekman, Xandra O. Breakefield, and Killian P. O'Brien

Supplemental Figure 1: Quantification of cellular communication with the split-Nanoluc construct. Related to Figure 1.

A.

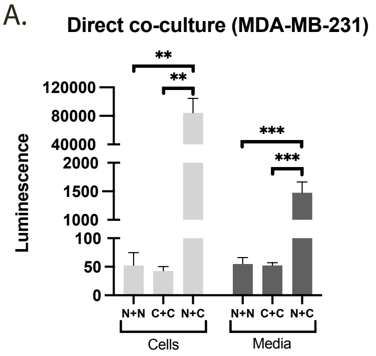

B.

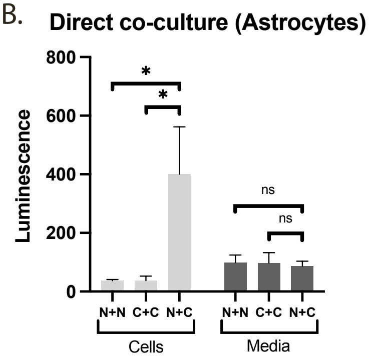

A) Direct co-culture of MDA breast cancer cells transduced with N65 or 66C constructs. Student's independent t-test, **: p<0.01, ***: p<0.001. Related to Figure 1

B) Direct co-culture of astrocytes transduced with either N65 of 66C construct. Student's independent t-test, ns: not significant, *: p<0.05. Related to Figure 1

C.

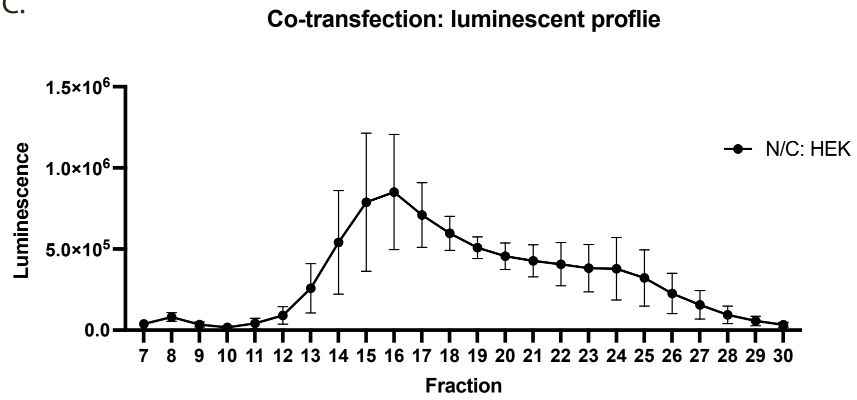

C) Media from HEK cells transfected with both N65 and 66C constructs separated via size exclusion chromatography. FMZ was added and signal recorded after 3 minutes. Related to Figure 1

Supplemental Figure 2: Extracellular vesicle mediated communication with the split-Nanoluc construct. Related to Figure 2.

A.

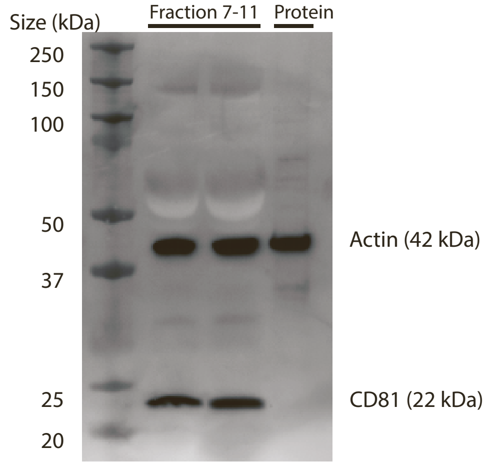

A) Western Blot of EV fractions and protein isolated from HEK293T cells. Stained for Actin and CD81. kDA: kilo Dalton. Related to Figure 2

B.

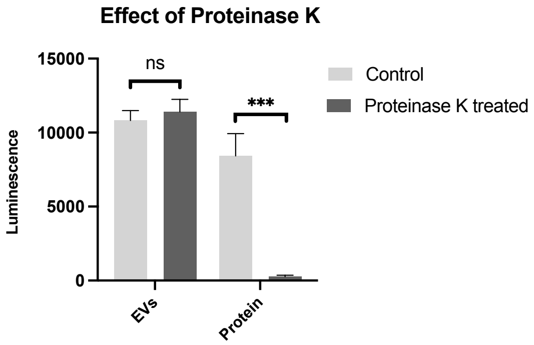

B) The effect of proteinase K treatment on the luminescent signal in EV and protein fractions. Student's independent t-test, ns: not significant, ***: p<0.001. Related to Figure 2.

C.

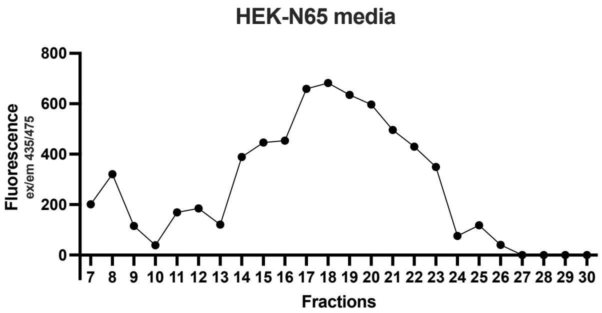

C) Fluorescent signal in different fractions derived via size exclusion chromatography from HEK cells transfected with the N65 construct. Related to Figure 2.

D.

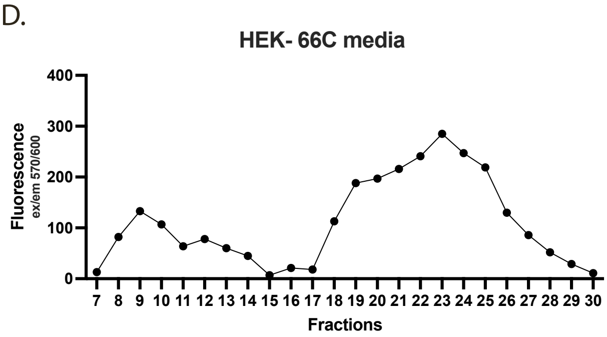

D) Fluorescent signal in different fractions derived via size exclusion chromatography from HEK cells transfected with the 66C construct. Related to Figure 2.

E.

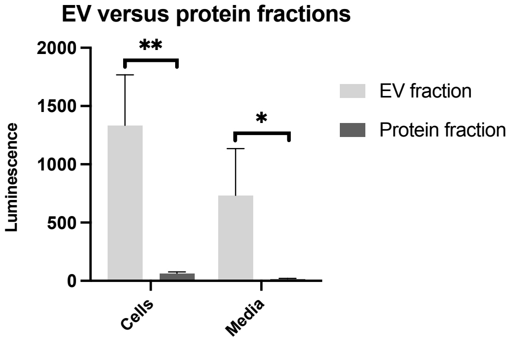

E) EVs and free proteins were isolated from N65 transfected HEK293T cells with size-exclusion chromatography and added to 66C transfected cells. The amount of EVs and protein added was normalized based on fluorescence. Nluc luminescence was then measured. Student's independent t-test, *: p<0.05, **: p<0.01. Related to Figure 2.
